# The G-Quadruplex Ligand Telomestatin Impairs Binding of Topoisomerase IIIα to G-Quadruplex-Forming Oligonucleotides and Uncaps Telomeres in ALT Cells

**DOI:** 10.1371/journal.pone.0006919

**Published:** 2009-09-09

**Authors:** Nassima Temime-Smaali, Lionel Guittat, Assitan Sidibe, Kazuo Shin-ya, Chantal Trentesaux, Jean-François Riou

**Affiliations:** 1 Laboratoire d'Onco-Pharmacologie, JE 2428, UFR de Pharmacie, Université de Reims Champagne-Ardenne, Reims, France; 2 Laboratoire de Régulations et Dynamique des Génomes, INSERM U565, CNRS UMR7196, Muséum National d'Histoire Naturelle USM503, CP26, Paris, France; 3 Biomedicinal Information Research Center, National Institute of Advanced Industrial Science and Technology, Biological Systems Control Team, Tokyo, Japan; University of Florida, United States of America

## Abstract

In Alternative Lengthening of Telomeres (ALT) cell lines, specific nuclear bodies called APBs (ALT-associated PML bodies) concentrate telomeric DNA, shelterin components and recombination factors associated with telomere recombination. Topoisomerase IIIα (Topo III) is an essential telomeric-associated factor in ALT cells. We show here that the binding of Topo III to telomeric G-overhang is modulated by G-quadruplex formation. Topo III binding to G-quadruplex-forming oligonucleotides was strongly inhibited by telomestatin, a potent and specific G-quadruplex ligand. In ALT cells, telomestatin treatment resulted in the depletion of the Topo III/BLM/TRF2 complex and the disruption of APBs and led to the segregation of PML, shelterin components and Topo III. Interestingly, a DNA damage response was observed at telomeres in telomestatin-treated cells. These data indicate the importance of G-quadruplex stabilization during telomere maintenance in ALT cells. The function of TRF2/Topo III/BLM in the resolution of replication intermediates at telomeres is discussed.

## Introduction

In vertebrates, the telomeres that cap chromosome ends are composed of tandem repeats of the sequence d(TTAGGG)n with a 3′ single-stranded extension (G-overhang) associated with six protein factors (TRF1, TRF2, RAP1, TIN2, TPP1 and POT1) that form a protecting complex (shelterin) essential for genome stability [1]. Telomeres shorten at each round of cell division and cellular mechanisms that counteract this loss confer indefinite proliferation potential characteristic of cancerous cells [2]. Two mechanisms have been reported in the maintenance of telomere length. The first requires a specialized enzyme, called telomerase, which is able to copy, as a reverse transcriptase, the short TTAGGG motif at the 3′ end of telomeres; the activity of telomerase is tightly controlled by shelterin [2]. The second mechanism involves recombination between telomeres, a mechanism known as Alternative Lengthening of Telomeres (ALT) [3]. ALT cells are characterized by the absence of telomerase activity, heterogeneous telomere length and the presence of nuclear foci termed ALT-associated PML bodies (APBs) that contain telomeric DNA, telomeric proteins TRF1 and TRF2, and DNA recombination/repair proteins [3].

*In vitro*, DNA oligonucleotides composed of the G-overhang DNA sequence may adopt a four-stranded structure called a G-quadruplex. Both direct and indirect evidence suggests that G-quadruplexes are also present in eukaryotic cells and that formation of this structure must be tightly regulated to allow DNA replication and cell division (for a review, see [4]). The binding of specific ligands to the telomeric G-quadruplex is a strategy to alter telomere functions and to inhibit cell growth. A number of G-quadruplex ligands have been synthesized during the last decade [5]. These compounds were initially derived from DNA intercalators [6] and now present selective binding properties to G-quadruplexes relative to duplex or single-stranded DNA [5]. The initial therapeutic rationale for use of these compounds in treatment of cancer was that inhibition of telomerase activity would inhibit unchecked cell proliferation [7], [8], but several pieces of evidence suggest that these ligands must be considered as telomere targeting agents rather than simple telomerase inhibitors [9]–[11].

The natural product telomestatin, is one of the most potent and selective G-quadruplex binding small molecules known [12]–[14]. Treatment with telomestatin impairs telomere replication and provokes the release of POT1 and TRF2 from telomeres, which results in loss of single- and double-stranded telomeric DNA and inhibits growth of tumor cells [15]. However, little is known about the effect of this compound or other G-quadruplex ligands on telomerase-negative ALT cell lines, except that telomestatin inhibits cell proliferation[8], [13], [16], [17].

G-quadruplex ligands also inhibit *in vitro* the activity of RecQ helicases WRN and BLM [18]. These helicases are associated with APBs in ALT cells and interact with shelterin components [19]–[21]. BLM is known to interact with Topoisomerase IIIα (Topo III) and two other proteins with OB-fold domains, RMI1 and RMI2, to form a RTR complex (RecQ/Topo III/RMI) essential to maintenance of genome stability through its function in the resolution of recombination intermediates (for recent reviews see [22], [23]). In this complex, Topo III and BLM cooperate to convert double Holliday junctions (DHJ) to decatenated products *in vitro* [24]. RMI1 guides the binding of Topo III [25]–[27] and RMI2 regulates other protein-protein interactions in the complex [28], [29]. The Topo III/BLM complex may function as a repair complex in response to replication defects and may restart stalled replication forks [30]–[32]. Following camptothecin treatment, a phosphorylated form of BLM dissociates from the Topo III/BLM complex at its PML storage sites and accumulates with γ-H2AX at damage sites during replication [33].

Topo III belongs to the Type IA DNA topoisomerase subfamily, which is conserved among different organisms; it removes highly negative supercoils where single-stranded DNA is exposed [34]. *In vitro* this access can be provided by altering the secondary structure of the substrate using hyper-negative supercoiling or by the addition of single-stranded binding protein (RPA, RMI1) to assist Topo III binding [27]. Structural studies of *E. coli* Topo III revealed that the protein first recognizes the presence of single-stranded DNA through a specific DNA binding region and then induces protein rearrangements that activate the tyrosine catalytic region [35]. This ensures the recognition of the correct type of DNA by the active site and explains the requirement for cofactors to generate single-stranded DNA.

Due to the diversity of complex structures that may form at telomere ends during fork progression, there is a requirement for factors that modulate topology, t-loop formation and resolution [36], [37]. Among these factors, Topo III is an essential telomeric-associated component in ALT cells [38], [39]. Topo III interacts with telomeric DNA and forms a complex with TRF2 and BLM at telomeres in ALT cells [39]. Inhibition of Topo III expression by siRNA reduced ALT cell survival, but did not affect telomerase positive cell lines. Moreover, repression of Topo III expression in ALT cells induced telomere uncapping associated with reduced levels of TRF2 and BLM proteins [39]. This data suggested that the Topo III/BLM/TRF2 complex plays a role in the resolution of topological intermediates that arise during telomere recombination [39].

To examine whether Topo III binding at telomeric sequences and its localization at APBs is modulated by G-quadruplex formation, we have used the selective G-quadruplex ligand telomestatin. We found that Topo III binding is strongly inhibited by stabilization of G-quadruplex structures and that this ligand induces the disruption of APBs in ALT cells. This effect mimics the depletion of Topo III by RNA interference as it induces the depletion of the Topo III/BLM/TRF2 complex and results in telomere uncapping as evidenced by DNA damage at telomeres. We suggest that telomere maintenance in ALT cells may be particularly sensitive to G-quadruplex formation due to the need for the Topo III/BLM/TRF2 complex to resolve or modulate intermediate recombination/replication structures at telomeres.

## Materials and Methods

### Topo III and POT1 binding assays

Topo IIIα was purified according to a previously published procedure [40]. Purified recombinant hPOT1, prepared after baculovirus expression, was a generous gift from Dr. D. Gomez (Institut de Pharmacologie et de Biologie Structurale, Toulouse, France).

The oligonucleotides used in the study were:

21G: 5′-GGGTTAGGGTTAGGGTTAGGG-3′

21G mu3: 5′-GGCTTACGGTTAGCGTTAGGG-3′

Wt26: 5′-TTAGGGTTAGGGTTAGGGTTAGGGTT-3′

Pu22myc: 5′-GAGGGTGGGGAGGGTGGGGAAG-3′

Pu22mu: 5′-GAGGGTGAAGAGGGTGGGGAAG-3′

24C: 5′-CCCTAACCCTAACCCTAACCCTAA-3′

S310: 5′-CCAGCTCTGCTTTGCATCTTT-3′

Oligonucleotides were labelled at the 5′ end with [γ-^32^P]-ATP using T4 Polynucleotide Kinase (New England BioLabs®).

Electrophoretic mobility shift assays using Topo III or hPOT1 were performed in 10 µL of the following solution: 50 mM HEPES (pH 7.9), 100 mM NaCl, 0.1 mM EDTA, 4% w/v sucrose, 2% v/v glycerol, 0.1 mg/mL BSA, 0.02% w/v bromophenol blue, 20 nM labelled oligonucleotide and Topo III (50 nM) or hPOT1 (30 nM). For competition assays, the indicated concentrations of unlabelled oligonucleotides (1, 3, 10, 30, 100, 300, or 1000 nM) were added to the reaction mixture. For binding inhibition assays, the indicated concentrations of telomestatin were added in a volume of 1 µL. Telomestatin was prepared at 5 mM in 1∶1 DMSO/MetOH and was dissolved to 100 µM in DMSO and further with water. The reaction mixture was incubated at room temperature for 30 min. Each individual mixture was separated immediately by electrophoresis on 1% agarose gels in 0.5X Tris-Borate-EDTA buffer. The gels were run at 80 V for 45 min, dried on Whatman DE81 paper, and visualized by a phosporimager (Typhoon 9210, Amersham). Data was analyzed using ImageQuant software (Amersham) and results were expressed as the ratio between the initial fraction of DNA bound to Topo III (f_0_) and the fraction of DNA bound to Topo III in the presence of the competitor (f) as a function of the competitor concentration in abscissa. Values correspond to the mean value of three independent experiments ± SD.

### Cell culture

The MRC5-V1/YFP-Topo III cell line is a stable clone of the ALT cell line MRC5-V1 transfected with YFP-Topo III [39] and was grown in Dulbecco's modified Eagle's medium (GIBCO/Invitrogen) supplemented with 10% (v/v) fetal bovine serum (FBS; Invitrogen), 10 mM L-glutamine, and 400 µg/mL of geneticin. After transfection, cells were selected for 15 days with 400 µg/mL geneticin and cultures were sorted by FACS analysis and further grown in the presence of 400 µg/mL of geneticin.

### Antibodies

Primary antibodies used in this work were raised against PML (PG-M3, mouse, sc-966 Santa Cruz Biotechnology), TRF2 (4A794, mouse, Upstate Biotechnology), BLM (C18, sc-7790 Santa Cruz Biotechnology), β-actin (clone AC-15, mouse, Sigma), active caspase-3 (IMG-144, mouse, Imgenex), γ-H2AX (mouse, Upstate Biotechnology), TRF1 (C19, goat, sc-1977 Santa Cruz Biotechnology), and Topo IIIα (clone D6, rabbit (Wu et al., 2000)). Secondary antibodies used for western experiments were goat anti-rabbit and anti-mouse HRP conjugates (Upstate Biotechnology) and donkey anti-goat HRP conjugates (Abcam). For immunofluorescence experiments, goat anti-mouse Alexa fluor 488 and 568 as well as a goat anti-rabbit Alexa fluor 568 (Invitrogen) were used. Immunoblotting was performed according to [41].

### G-overhang hybridization assays

The nondenaturing hybridization assay to detect the 3′ telomeric G-overhang was performed as described previously using the (AATCCC)_4_ oligonucleotide [42].

### Immunofluorescence

Cells were plated in 6-well culture plates on glass coverslips. After drug treatment, cells were washed with 1X PBS (pH 7.2), fixed with 4% paraformaldehyde, and permeabilized with 20 mM Tris-HCl (pH 8.0), 50 mM NaCl, 3 mM MgCl_2_, 300 mM sucrose and 0.5% v/v Triton X-100 for 15 min at room temperature. Cells were then washed twice with PBS (pH 7.2). Cells were blocked with 1% BSA in 1X PBS for 1 hour and incubated with primary antibodies for 1 hour to 2 hours at room temperature. After washing with 1X PBS proteins of interest were detected by incubation for 30 min with fluorescently label secondary antibodies, then washed with 1X PBS. The nuclear DNA was stained with 0.1 µg/mL DAPI in PBS (pH 7.2) for 4 min. Cells were mounted in Shandon Immu-Mount medium (Thermo Scientific). Samples were observed with a DMR Leica microscope and images were captured with a Cool Snap HQ camera (Roper Scientific) controlled by Metamorph software (Roper Scientific). Final images are composed of arithmetic stacks of 15–25 deconvolued images, each 0.2 µM in z-step. Stacks of 15–30 images (16 bit grayscale) were acquired with a z-step of 0.2 µm with low illumination intensity. For the quantification of co-localizations, 50 nuclei were analyzed and results represent the mean ± SD relative to YFP-Topo III or TRF1 foci, as indicated.

## Results

### Human Topo III binds to G-quadruplex but prefers single-stranded structures

**Figure 1:**
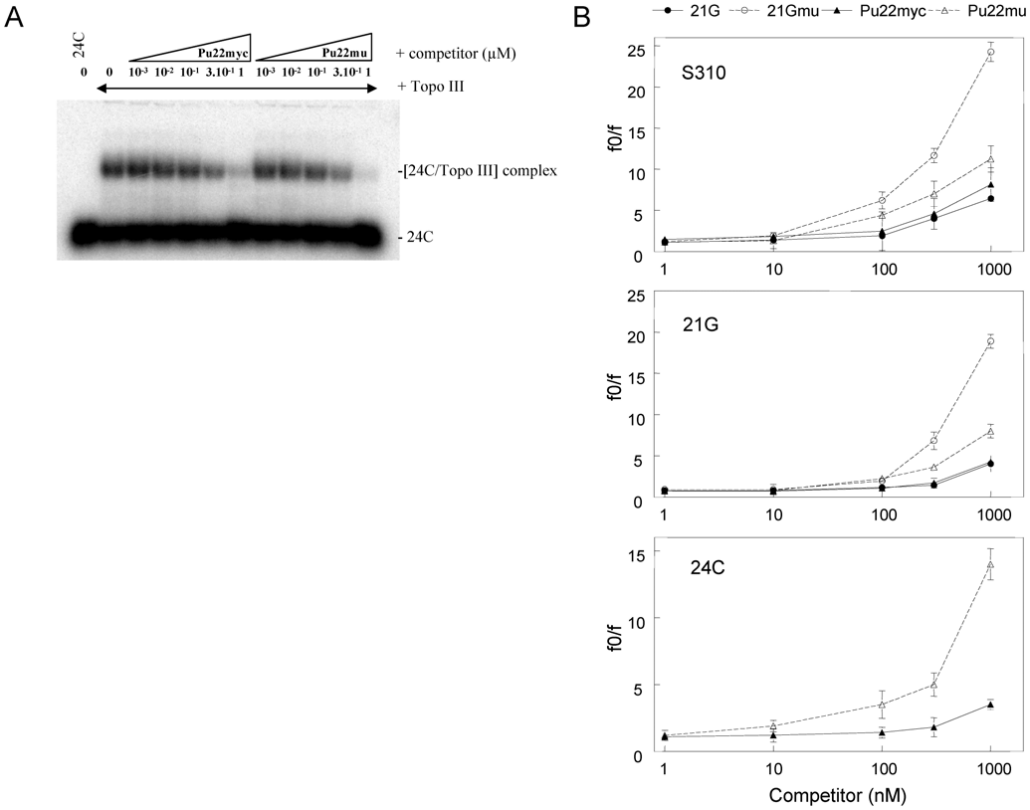
Topo III preferentially binds to single-stranded oligonucleotides when compared to G-quadruplex-forming sequences. A, Competition experiment for Topo III (50 nM) binding to [^32^P]-labeled 24C oligonucleotide (20 nM) in the presence of either unlabeled Pu22myc or Pu22mu (see sequences in Material and Methods) at the indicated concentrations (in µM). B, Quantification of the competition experiments for Topo III binding to [^32^P]-labeled S310 (top), 21G (middle), and 24C (bottom) oligonucleotides (at 20 nM) with increasing concentrations (1 to 1000 nM) of unlabeled 21G (closed circles), 21Gmu (open circles), Pu22myc (closed triangles), and Pu22mu (open triangles) oligonucleotides. Results correspond to the mean ± SD of three determinations. f_0_ is the initial fraction of DNA bound to Topo III and f the fraction of DNA bound to Topo III at a determined concentration of the competitor.

Purified recombinant Topo III interacts with single-stranded DNA, including the telomeric G-overhang sequence [39]. Since telomeric G-overhang is prone to form G-quadruplexes, we have examined whether the presence of guanines repeats influences the DNA binding of Topo III. A direct binding assay for Topo III to the telomeric G-overhang or to a mutated sequence unable to form G-quadruplex indicated that Topo III has a slight preference for single-stranded DNA that cannot fold into a G-quadruplex (result not shown). To quantify differences in binding, we have performed competition experiments with radioactively labeled single-stranded oligonucleotides (21G, S310 and 24C), unlabeled DNA oligonucleotides able to form G-quadruplex (21G, Pu22myc) or unable to form G-quadruplex (21Gmu, Pu22mu) [43], [44]. The sequences of oligonucleotides are given in the Materials and Methods section. As an example, Figure 1A presents the analysis of the competition for Topo III binding to oligonucleotide 24C with either the G-quadruplex forming Pu22myc or its mutant Pu22mu, which is unable to fold into G-quadruplex. A 3-fold higher concentration of Pu22myc than Pu22mu was needed to inhibit Topo III binding to single-stranded oligonucleotide 24C. Similar differences between G-quadruplex-forming and mutant oligonucleotides were obtained for Topo III binding to 21G and S310 oligonucleotides (Figure 1B). The IC_50_s for competition with oligonucleotides that cannot form G-quadruplex was 4.3 to 10.1-fold lower than for G-quadruplex forming oligonucleotides in these experiments (Figure 1B), indicating that Topo III prefers single-stranded oligonucleotides relative to G-quadruplex forming oligonucleotides.

### Telomestatin inhibits Topo III binding to the telomeric G-overhang

**Figure 2:**
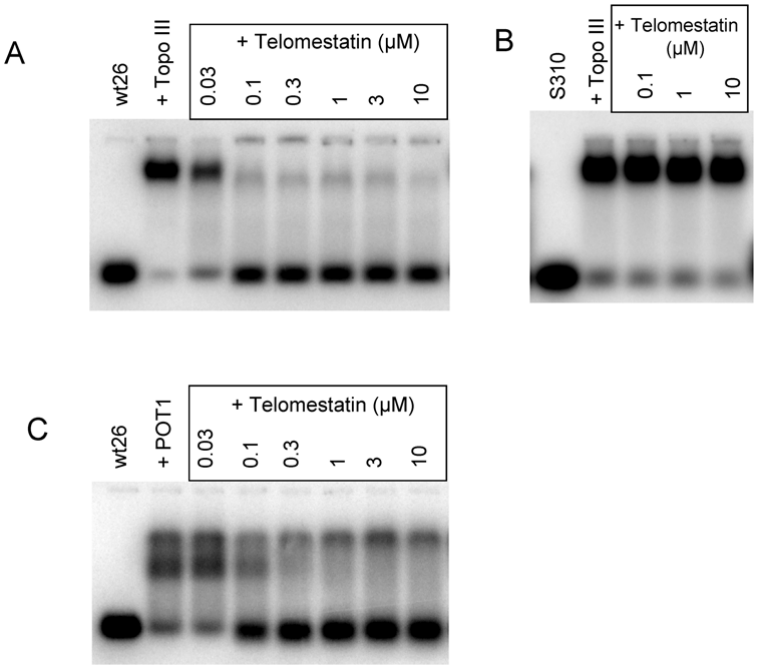
Telomestatin inhibits Topo III and POT1 binding to telomeric G-overhangs. A, Bandshift assay using purified Topo III (50 nM) and [32P]-labeled Wt26 in the presence of the indicated concentrations of telomestatin. B, Bandshift assay using purified Topo III (50 nM) and [32P]-labeled S310 (20 nM) in the presence of the indicated concentrations of telomestatin. C, Bandshift assay using POT1 (30 nM) and [32P]-labeled Wt26 (20 nM) in the presence of the indicated concentrations of telomestatin. Telomestatin inhibits Topo III and POT1 binding to Wt26, which forms a G-quadruplex, but not to S310, which does not.

**Figure 3:**
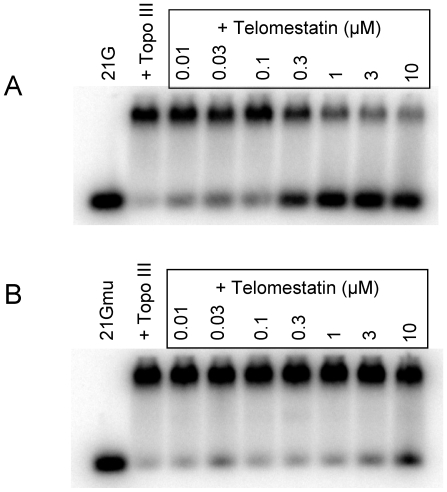
Inhibition of Topo III binding to telomeric sequence by telomestatin is due to G-quadruplex formation. A, Bandshift assay of purified Topo III (50 nM) to [32P]-labeled 21G (20 nM) in the presence of the indicated telomestatin concentrations. B, Bandshift assay of purified Topo III (50 nM) to [32P]-labeled 21Gmu (20 nM) in the presence of the indicated telomestatin concentrations. Telomestatin had no effect on binding of Topo III to the telomeric mutant sequence 21Gmu, which cannot form a G-quadruplex.

We next determined whether ligands that stabilize G-quadruplex formation interfere with the binding of Topo III to the telomeric G-strand. Telomestatin was isolated from *Streptomyces annulatus* and potently and selectively binds to and stabilizes telomeric G-quadruplexes [12]–[14]. In these experiments, we used the Wt26 oligonucleotide previously shown to form a G-quadruplex [45]. In the presence of telomestatin, we observed a strong inhibition of Topo III-Wt26 complex as detected by bandshift experiments (Figure 2A). Slight inhibition was observed at 0.03 µM telomestatin and no complex was observed at 0.1 µM; the IC_50_ was 60 nM. In contrast, telomestatin (up to 10 µM) had no effect on Topo III binding to the single-stranded oligonucleotide S310 (Figure 2B). Telomestatin efficiently inhibited Topo III binding to G-quadruplex forming 21G (IC_50_ equal to 300 nM) but not to the mutant sequence 21Gmu (IC_50_ >10 µM) (Figure 3A and B). We obtained similar results using other G-quadruplex ligands such as the pyridine dicarboxamide derivative 360A and the steroid derivative funtumine guanylhydrazone [17], [46] (results not shown). We concluded that the stabilization of the G-quadruplex structure by specific ligands such as telomestatin efficiently impaired Topo III binding.

We previously reported that telomestatin inhibits the binding of POT1 to the telomeric G-overhang in a coupled transcription and translation assay [42]. The use of purified recombinant POT1 protein under bandshift conditions indicated that telomestatin also inhibited the binding of POT1 to longer oligonucleotides (5′-G_3_(T_2_AG_3_)_7_-3′, IC_50_ of 500 nM [47]) and to Wt26 (IC_50_ of 100 nM) (Figure 2C). Interestingly, these values are in the same range that of telomestatin for inhibition of Topo III binding to telomere oligonucleotides (60–300 nM). Since telomestatin was shown to uncap POT1 from telomeres in cell culture [42], [48], [49], we speculated that telomestatin may also alter recruitment of Topo III to telomeres.

### Treatment with telomestatin reduces levels of Topo III, TRF2, and BLM and disorganizes APBs in MRC5-V1 ALT cells

**Figure 4:**
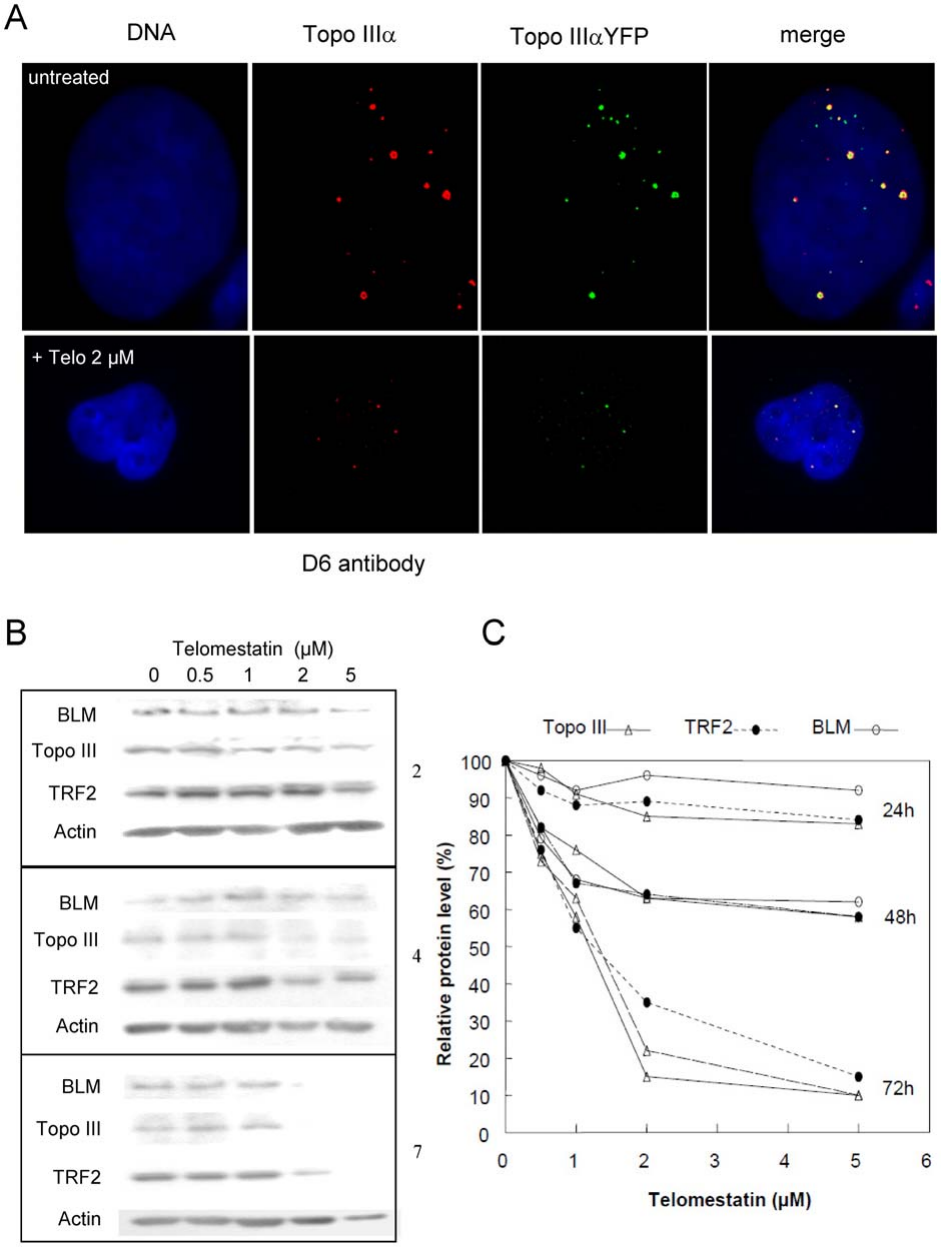
Effects of telomestatin on Topo III signals and Topo III/TRF2/BLM protein levels in MRC5-V1/YFP-Topo III cells. A, Representative images of untreated MRC5-V1/YFP-Topo III cells and cells treated with 2 µM telomestatin for 48 h; Topo III was detected by fluorescence of YFP-tagged Topo III (green) or by immunofluorescence using D6 antibody (red). Dapi staining of DNA is shown in blue. Telomestatin induced a decrease of Topo III foci number and intensity. B, Cells were treated with telomestatin (0.5 to 5 µM) for 24, 48, or 72 h and Topo III, BLM, TRF2, and actin protein levels were detected by western blot. Telomestatin induced a dose-dependent decrease in Topo III, TRF2, and BLM. C, quantification of the western blot experiment. Result were normalized relative to actin protein levels and expressed relative to control untreated cells, defined as 100%.

Topo III co-localizes with telomeric proteins at APBs in ALT cells [39]. To examine the effects of telomestatin treatment on the recruitment of Topo III to these nuclear bodies in cultured cells, we stably transfected a YFP-tagged Topo III construct into MRC5-V1 ALT cells [39]. Using a D6 antibody directed against Topo III, it was shown that the tagged protein localizes at the same sites as does the endogenous protein ([39], [50], see also Figure 4A). Therefore, it can be used to investigate the cellular effect of telomestatin on Topo III localization. The YFP-Topo III fluorescence signal, which mostly corresponded to large nuclear foci, was decreased after telomestatin treatment (Figure 4A). The immunofluorescence signal of the D6 antibody was also decreased in telomestatin-treated cells, indicating that telomestatin alters Topo III protein levels (Figure 4A). Further analysis by western blot showed that Topo III protein levels were decreased by telomestatin treatment in a dose- and time-dependent manner (Figure 4B). After 72 hours of treatment with 2 µM telomestatin, we observed a nearly complete loss of Topo III protein (relative to actin). Since Topo III, BLM, and TRF2 form a complex in ALT cells [39], we next investigated if telomestatin treatment had an effect on the BLM and TRF2 protein levels. As shown in Figure 4B&C, the Topo III decrease was associated with a significant decrease in BLM and TRF2 protein levels, but there was not significant induction of apoptosis as measured by caspase 3 cleavage (result not shown). This suggests that, like siRNA inhibition of Topo III translation [39], telomestatin triggers Topo III/TRF2/BLM complex degradation in ALT cells.

**Figure 5:**
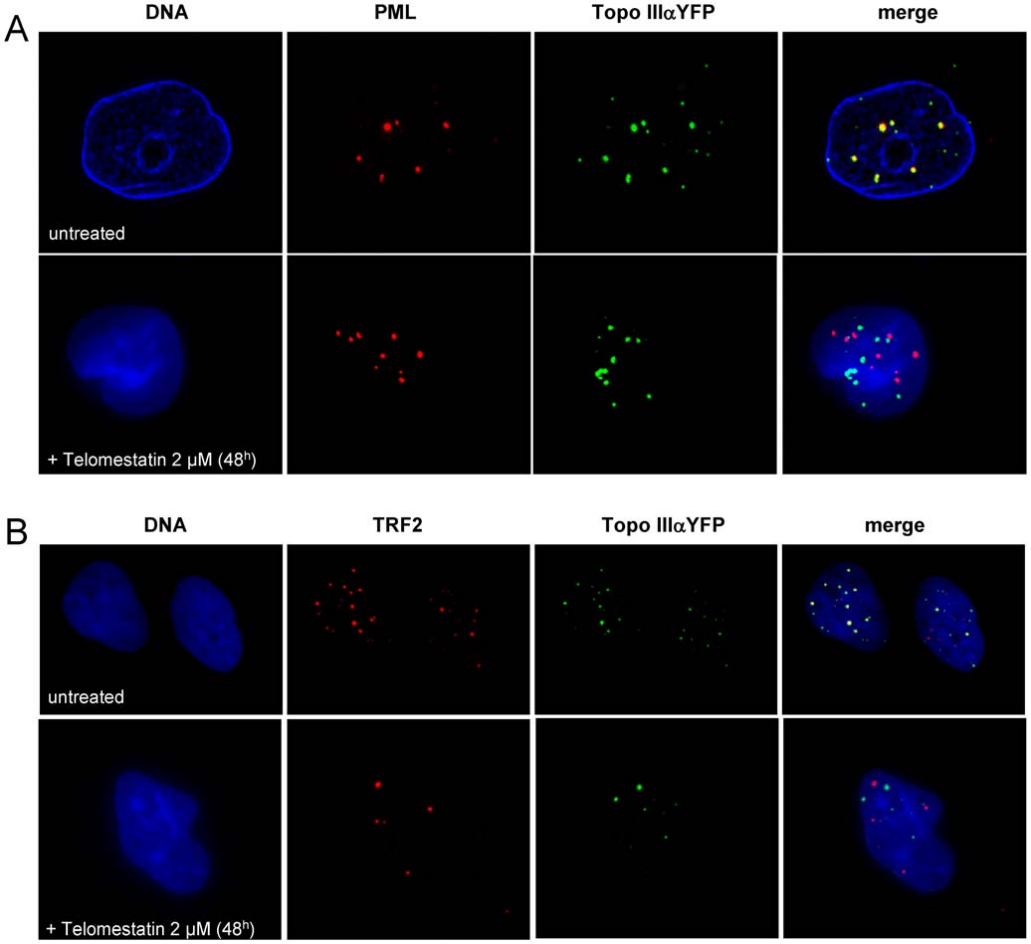
Telomestatin induces a delocalization of Topo III from PML and TRF2. Representative images are of untreated MRC5-V1/YFP-Topo III cells or cells treated for 48 h with 2 µM telomestatin. A, Topo III detected by fluorescence of YFP-tagged Topo III (green) and PML detected by immunofluorescence (red). Dapi staining of DNA is shown in blue. B, Topo III detected by fluorescence of YFP-tagged Topo III (green) and TRF2 detected by immunofluorescence (red). Dapi staining of DNA is shown in blue. The extent of co-localization of Topo III and PML and of Topo III and TRF2 is decreased by telomestatin treatment relative to that in untreated cells.

**Figure 6:**
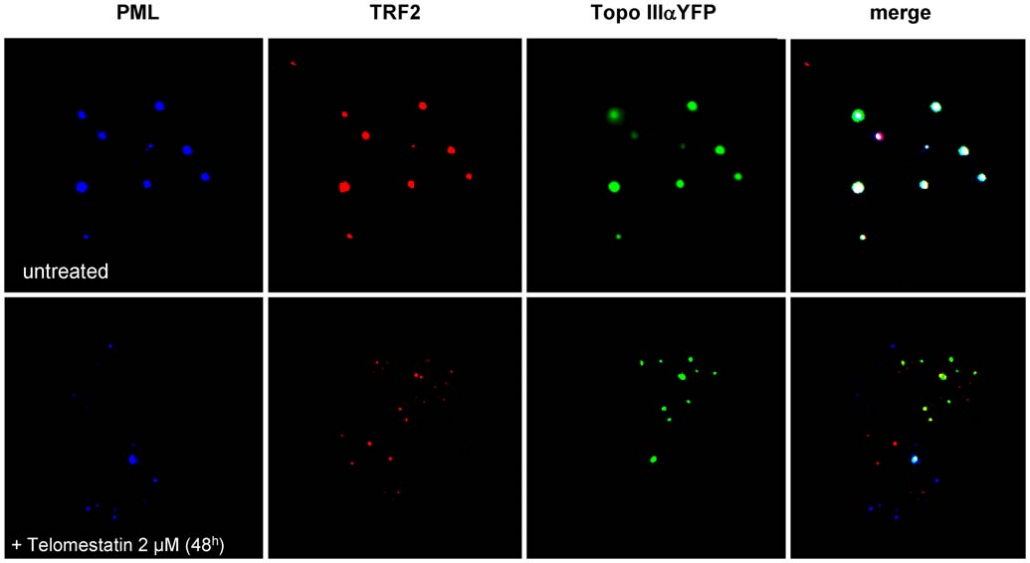
Co-localization of Topo III with PML and TRF2 is inhibited by telomestatin treatment. Representative images are of untreated MRC5-V1/YFP-Topo III cells and cells treated with 2 µM telomestatin for 48 h; YFP-tagged Topo III is shown in green, TRF2 is shown in red (detected by immunofluorescence), and PML is shown in blue (detected by immunofluorescence).

**Figure 7:**
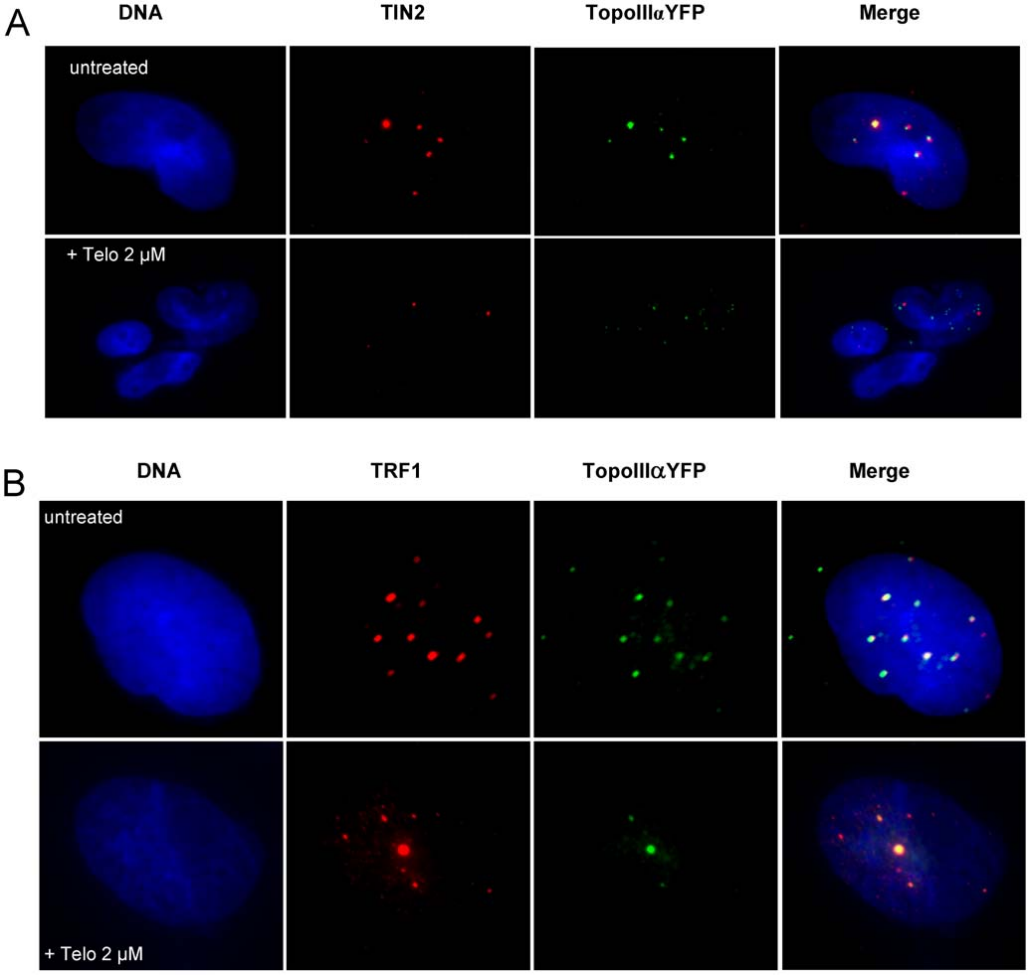
Telomestatin induces a de-localization of Topo III from TIN2 and TRF1. Representative images are of untreated MRC5-V1/YFP-Topo III cells or cells treated for 48 h with 2 µM telomestatin. A, Topo III was detected by fluorescence of YFP-tagged Topo III (green) and TIN2 was detected by immunofluorescence (red). Dapi staining of DNA is shown in blue. B, Topo III was detected by fluorescence of YFP-tagged Topo III (green) and TRF1 was detected by immunofluorescence (red). Dapi staining of DNA is shown in blue. The amount of co-localization between Topo III and TIN2 or Topo III and TRF1 observed in untreated cells was decreased by telomestatin treatment.

**Figure 8:**
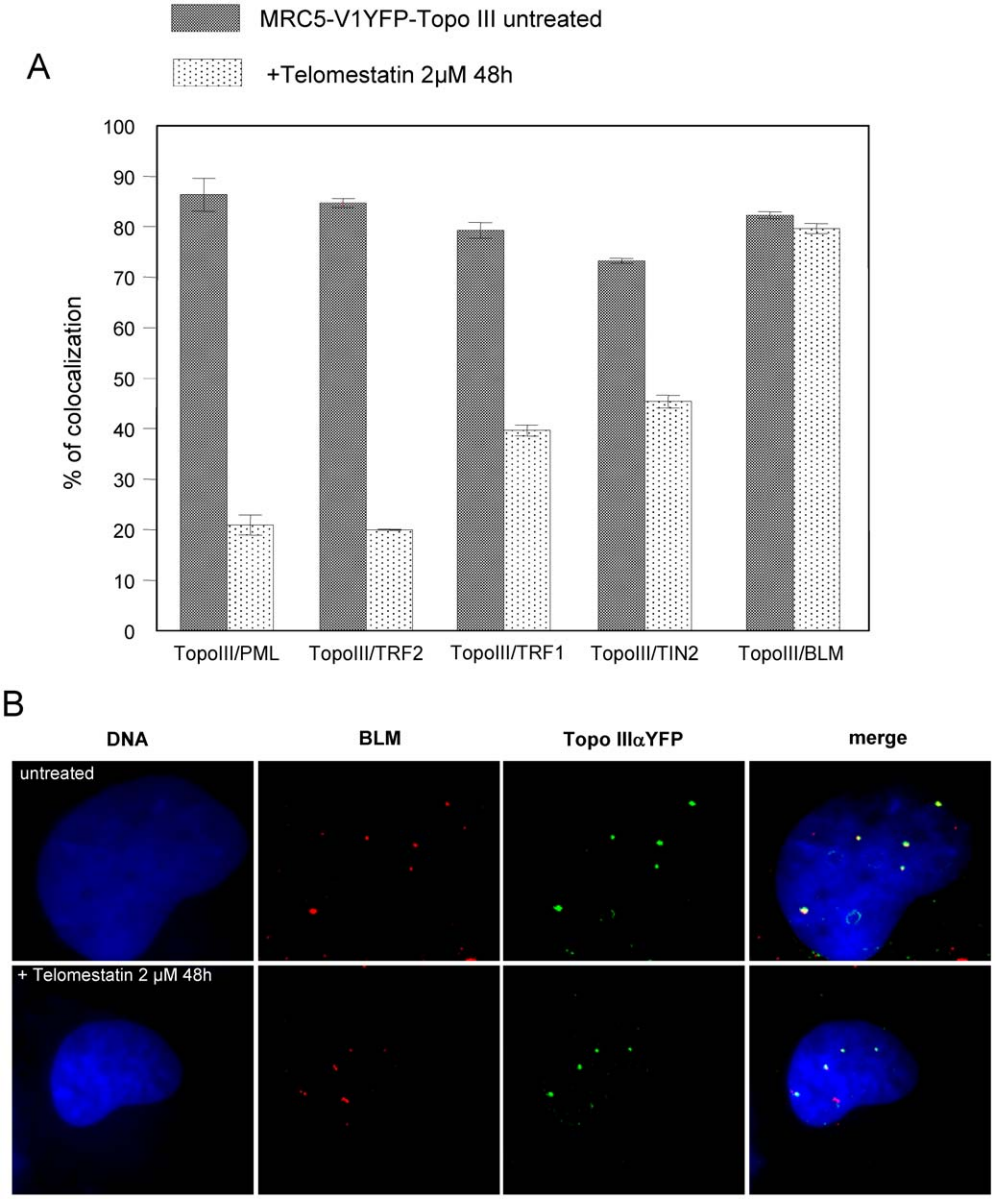
Telomestatin induces a delocalization of Topo III from PML and shelterin components but not from BLM. MRC5-V1/YFP-Topo III cells were left untreated or were treated for 48 h with 2 µM telomestatin. A, Quantification of the co-localization between Topo III and PML, TRF2, TRF1, TIN2, and BLM in untreated and treated cells. Results are expressed as the relative percentage of Topo IIIα foci that co-localize with PML, TRF2, TRF1, TIN2, or BLM foci and were determined by analysis of more than 50 nuclei. B, Topo III was detected by fluorescence of YFP-tagged Topo III (green) and BLM was detected by immunofluorescence (red). Dapi staining of DNA is shown in blue. The co-localization of Topo III and BLM was not altered by telomestatin treatment.

To further analyze the effect of telomestatin on organization of APBs in MRC5-V1/YFP-Topo III cells, we treated cells with 2 µM telomestatin for 48 h, because these conditions measurably reduce Topo III, TRF2 and BLM protein levels but cells remain viable. This allowed us to perform nuclear localization studies by microscopy. Microscopic examination of treated cells showed important changes in the nuclear organization of YFP-Topo III at APBs. Telomestatin reduced the co-localization of YFP-Topo III with PMLs (Figure 5A) and with TRF2 (Figure 5B). Double staining with anti-TRF2 and with anti-PML antibodies allowed us to visualize these APBs components in MRC5-V1/YFP-Topo III cells. After telomestatin treatment, YFP-Topo III, TRF2, and PML were observed in separate foci (Figure 6). Similar loss of co-localization was observed between YFP-Topo III and two other factors of the shelterin complex, TIN2 and TRF1 (Figure 7 A and B). After telomestatin treatment, only 20% of YFP-Topo III co-localized with PML and TRF2 and only about 45% of YFP-Topo III co-localized with TRF1 or TIN2 (Figure 8A). Interestingly, telomestatin did not qualitatively alter the co-localization between YFP-Topo III and BLM (Figure 8A and B); however, BLM protein levels decreased after telomestatin treatment as shown by western-blot (Figure 4B), suggesting that telomestatin does not alter the interaction between these two proteins. These results indicate that the G-quadruplex ligand telomestatin decreases the amount of Topo III/BLM/TRF2 complex present in cells and disrupts the formation of APBs.

Co-immunoprecipitation experiments were also performed to study whether the interaction of TRF2 or BLM with Topo III is altered by telomestatin treatment. In the complex precipitated by the Topo III D6 antibody, we detected TRF2 and BLM by immunoblotting (Figure S1 and [39]). After 48 hours of treatment with 2 µM telomestatin, we observed a nearly complete loss of TRF2 protein associated with Topo III. In contrast, BLM associated with Topo III is not affected by telomestatin treatment. These results confirm the immunofluorescence observations.

### Telomestatin induces a massive DNA damage response at telomeres in ALT cells

**Figure 9:**
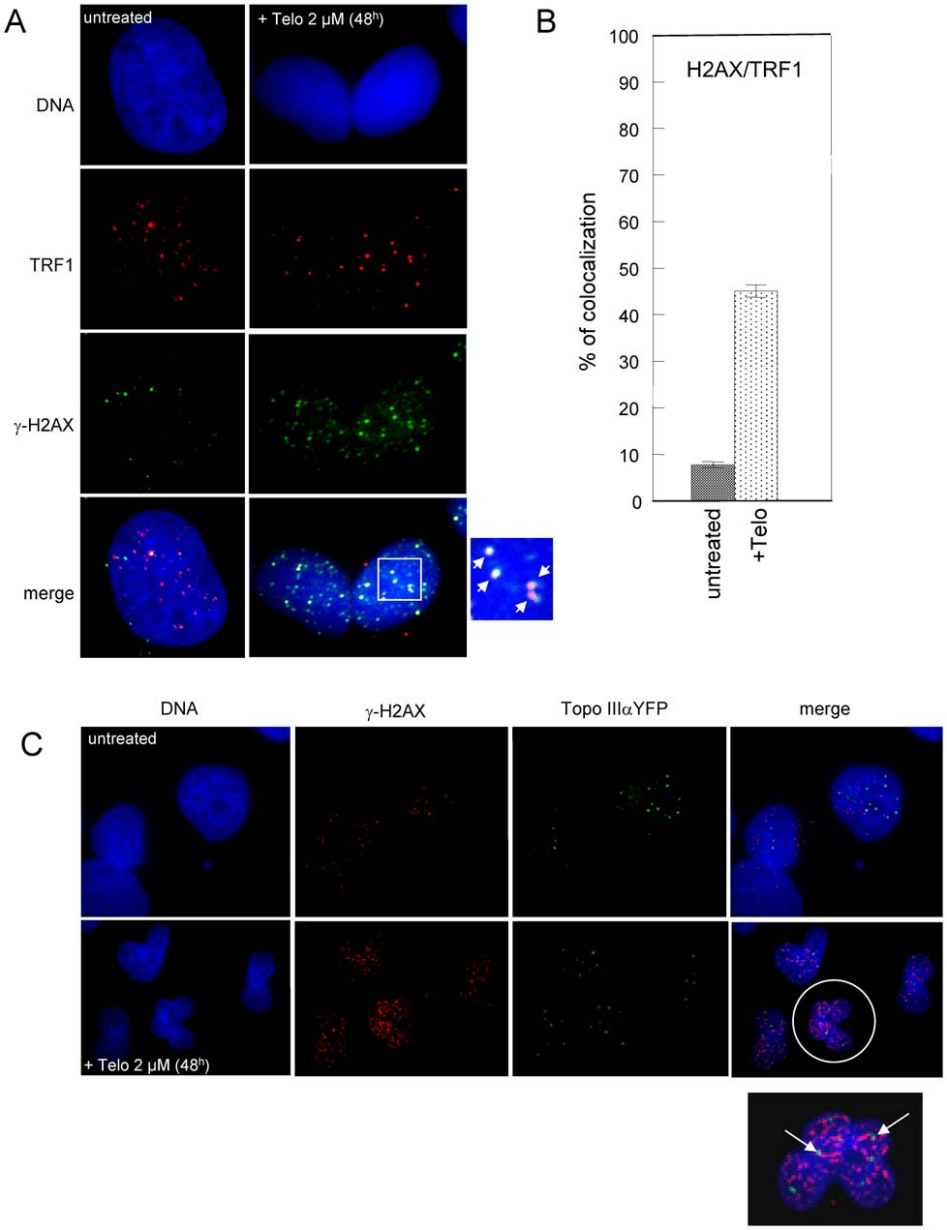
DNA damage response after telomestatin treatment of MRC5-V1/YFP-Topo III cells. A, Cells treated for 48 h with 2 µM telomestatin or left untreated were examined for γ-H2AX foci (green) that co-localized with TRF1 (red). B, Quantification of the DNA damage foci that co-localized with TRF1. Results are expressed as the percentage relative to γ-H2AX foci number and were determined by analysis of more than 50 nuclei. C, Cells treated for 48 h with 2 µM telomestatin or left untreated were examined for γ-H2AX foci (red) that co-localized with YFP-Topo III (green). In the magnification at the bottom the co-localization between γ-H2AX and Topo III is indicated by arrows.

**Figure 10:**
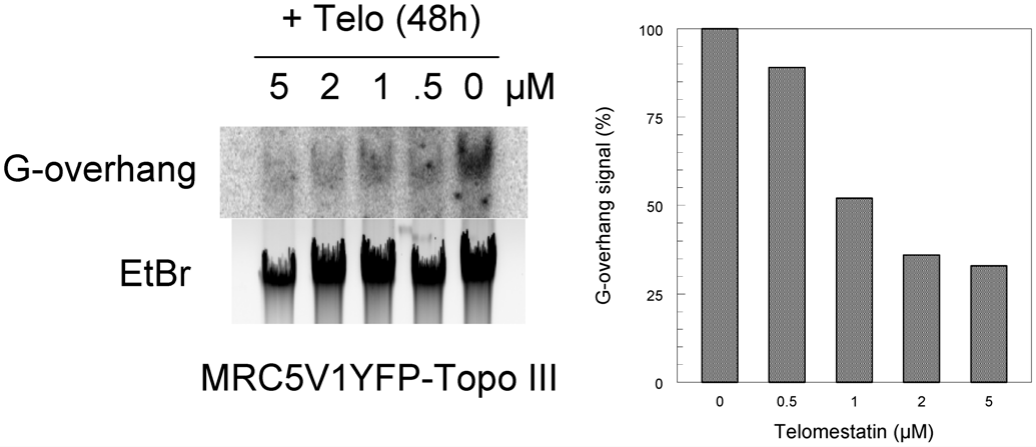
Effect of telomestatin on G-overhang signal. A, DNA was extracted from untreated MRC5-V1/YFP-Topo III cells or cells treated for 48 h with telomestatin at indicated concentrations. The G-overhang signal was evaluated by non-denaturing solution hybridization with a telomeric probe (AATCCC)_4_. The gel was also stained with ethidium bromide. B, Quantification of the experiment presented in A. The G-overhang hybridization signal was normalized relative to the ethidium bromide signal. Results are expressed as the percentage of G-overhang signal in control untreated cells, which was defined as 100%.

To further determine whether telomestatin-induced ABP disruption affected telomere integrity, we tested MRC5-V1/YFP-Topo III cells for DNA damage that co-localized with telomeres. Telomere dysfunction-induced foci (TIFs) can be detected due to the presence of γ-H2AX, a phosphorylated variant of histone 2A that associates with DNA double-strand breaks [51]. Cells treated with 2 µM telomestatin for 48 h were analyzed for γ-H2AX-containing foci that co-localized with TRF1 (Figure 9A and B). There was a background level of γ-H2AX foci in untreated MRC5-V1/YFP-Topo III cells of 14±6 foci/nucleus; only 8% (±2%) of these foci co-localized with TRF1. In the presence of telomestatin, we observed an increased number of γ-H2AX foci to 68±4 foci/nucleus and almost half co-localized with TRF1 (46±4%), suggesting that telomestatin triggers a DNA damage response that mostly takes place at telomeres. However, the analysis of treated cells foci that contain both YFP-Topo III and γ-H2AX indicated that most of the DNA damage response was not associated with Topo III foci (Figure 9C). We then evaluated the amount of G-overhang telomeric DNA signal in MRC5-V1/YFP-Topo III cells after treatment with telomestatin using solution hybridization experiments (Figure 10). A significant reduction of the hybridization was observed in telomestatin-treated cells (40% of the signal observed in untreated cells). This is in agreement with other reports where telomestatin alters telomeric G-overhang in telomerase-positive cell lines[41], [44], [46]. Together, these results indicate that telomestatin induces a massive DNA damage response at telomeres.

## Discussion

Telomestatin binds specifically to G-quadruplex structures. In cells with telomerase activity, this binding results in telomere uncapping and leads to the G-overhang degradation and the release of telomeric proteins (POT1 and/or TRF2). Telomere uncapping is associated with a DNA damage response on a subset of telomere ends (for a recent review, see [15]). Little was known about the effect of telomestatin or other G-quadruplex ligands in telomerase-negative ALT cell lines, except that cell proliferation is inhibited [8], [16], [17], [52]. The results discussed here indicate that telomestatin induces G-overhang degradation in MRC5-V1/YFP-Topo III cells, an effect also observed in another ALT cell line, WI38-VA13 cells (result not shown). Our data suggest that alteration of the conformation or length of the telomeric G-overhang is a relevant marker for telomestatin activity in both telomerase positive and ALT cells.

Interestingly, telomestatin provoked a massive DNA damage response at telomeres in ALT cells, a result that contrasts with previous finding for this compound in telomerase-positive cell lines where only a small fraction of DNA damage foci corresponded to TIFs [49], [53]. Either telomestatin-induced DNA damage mostly takes place in extra-telomeric regions where potential G-quadruplex forming sequences have been identified [4] or the G-quadruplexes stabilized by telomestatin may be tightly regulated in telomeric regions during telomere replication. Specific G-quadruplex resolvases may repair these G-quadruplex structures to avoid replication blocks and DNA damage accumulation. For example, the FANCJ helicase suppresses the DNA damage response induced by telomestatin in telomerase-positive cells [53].

**Figure 11:**
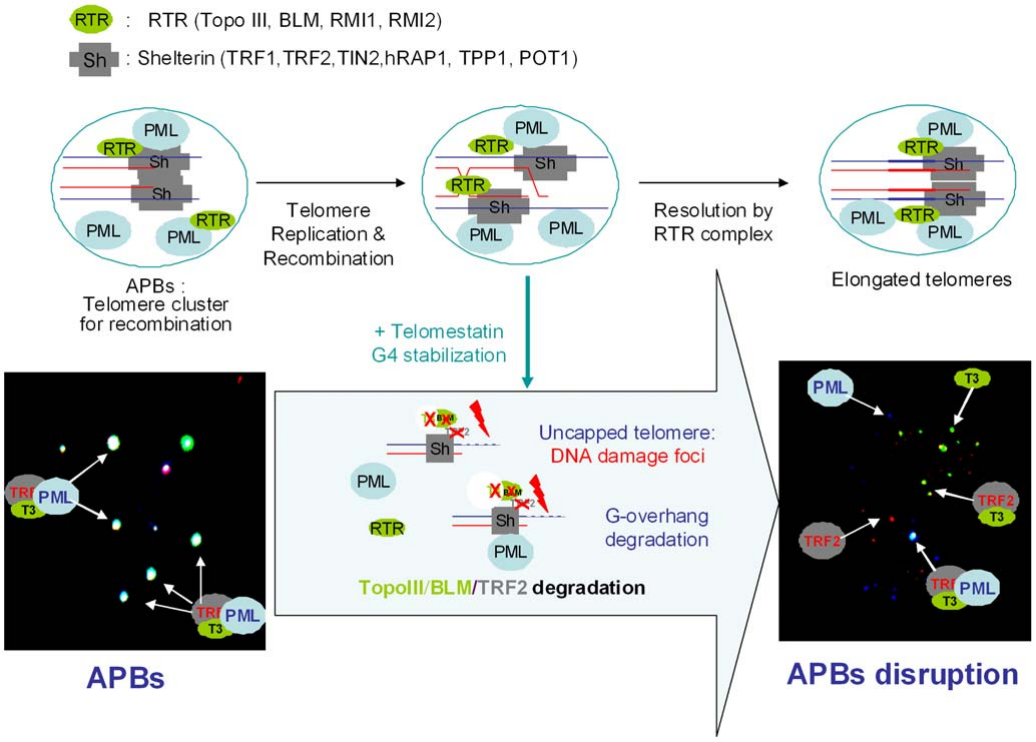
Model for the action of telomestatin in ALT cells. Short telomeres to be elongated are present in the APB platform and capped with proteins from the shelterin complex (Sh), including TRF1, TRF2, TIN2, TPP1, hRAP1 and POT1. The APBs also contains PML protein and proteins from the RTR complex (RTR), including Topo III, BLM, RMI1 and RMI2. Other recombination factors are not represented for sake of clarity. Telomere elongation takes place after homologous recombination (strand invasion); a possible mechanism for the resolution of the double Holliday junction occurring at the end of the recombination process involves Topo III/BLM recruitment by TRF2 at telomeres. In the presence of telomestatin, G-quadruplex stabilization induces APB disruption, which is a phenocopy of the siRNA-mediated Topo III deficiency, together with Topo III, BLM and TRF2 depletion, exposure of telomere ends to DNA damage (uncapping) and G-overhang degradation. A magnification of the image from Figure 6 is presented to illustrate the modification of the colocalization between PML, Topo III and TRF2.

The massive DNA damage response at telomeres in ALT cells after telomestatin treatment is accompanied by depletion of the Topo III/BLM/TRF2 complex and by telomere uncapping. These are the same effects observed after Topo III depletion by siRNA in ALT cells [39]. Telomestatin treatment also disrupted APBs, as evidenced by the segregation of Topo III, PML, and other shelterin components into separate foci (Figure 11). A close linkage between the formation of APBs, the presence of proteins from the shelterin complex and telomere maintenance by recombination has been suggested, although their structural organization is poorly understood [54], [55]. These specialized bodies contain recombination proteins that are thought to support the processes that maintain telomere length in the absence of telomerase activity. Recent evidence indicates that APBs provide a platform (called telomere clusters) where post-replicative telomere recombination intermediates are resolved [56]. These structures may maintain a spatio-temporal organization of telomeres needed for the completion of these reactions. Our data are consistent with a redistribution of unprotected telomere ends due to the absence of the telomere bouquet after telomestatin treatment.

The mechanistic details of the telomestatin-induced APB disruption are unclear. The depletion of several components of the shelterin complex, including TRF1, TRF2, TIN2 and RAP1 was previously shown to reduce formation of APBs [55]. Because telomestatin impairs the binding of POT1 and Topo III to the telomeric G-strand *in vitro*, the simplest explanation is that telomestatin disrupts the interactions to telomeres of telomere-binding proteins, inhibiting correct assembly of proteins on the APB platform. Although TRF2 does not directly bind to the telomeric G-overhang, G-quadruplex stabilization may induce indirect structural alterations at telomere ends or during replication, leading to the release and depletion of TRF2.

Several pieces of evidence indicate that TRF2 affects telomere structure and represses homologous recombination events at the t-loop [1]. The N-terminal domain of TRF2 was recently reported to introduce a torsional stress in telomeric DNA, to promote strand invasion into duplex DNA and to bind to Holliday Junction structures [37], [57]. We have proposed that Topo III might play a general role in ALT telomere physiology by resolving TRF2-mediated topological intermediates to modulate the t-loop formation or to modulate recombination processes [39]. It is likely that Topo III, which is essentially a nicking-closing enzyme that recognizes a single-stranded DNA region, catalyzes the resolution of the D-loop formed at t-loop or the double-Holliday junctions formed during telomere recombination (Figure 11).

The G-quadruplex stabilization by telomestatin may interfere with t-loop formation/dissolution or with Holliday junction resolution at telomere ends. In agreement, *in vitro* experiments indicated that telomestatin inhibited the strand invasion reaction of a G-strand oligonucleotide into a telomeric duplex suggesting that the ligand is able to block the t-loop formation (JF Riou, unpublished results). The specific difference between ALT cells and telomerase-positive cells is due to the respective presence or absence of APBs where recombination takes place. Therefore, the inhibition of the double-Holliday junction resolution by telomestatin might be more deleterious for telomere capping than the inhibition of the t-loop formation/resolution. However we cannot exclude the possibility that the resolution of t-loop formation in ALT cells represents a critical process. Our data provides a possible clue to the important effects of telomestatin at telomeres in ALT cells and further experiments aiming to determine whether Topo III controls recombination at telomeres in mammalian cells or results from the indirect loss of TRF2 triggered by the telomestatin treatment would be of great interest.

In conclusion, our results show that the G-quadruplex ligand telomestatin induces a telomere dysfunction in ALT cells, associated with APBs disruption and Topo III/BLM/TRF2 complex depletion. This emphasizes the interest of G-quadruplex ligands as potential therapeutics in tumor cells with alternative telomere maintenance mechanism.

## Supporting Information

Figure S1 Topo III/TRF2 complex is impaired in MRC5V1/YFP-Topo III cells treated with 2 µM telomestatin for 48 h. In contrast, Topo III/BLM complex is not modified by telomestatin treatment. Co-Immunoprecipitation was performed with D6 rabbit polyclonal antibody directed against Topo III as described previously [1]. Briefly, 2×10^7^ cells were lysed using RIPA buffer supplemented with 330 mM NaCl. The extract was precleared by Protein G Sepharose 4 Fast Flow and was mixed overnight with D6 anti-Topo III antibody. 100 µl of protein G-Sepharose beads were added to each sample, and beads were collected by centrifugation and washed three times with PBS buffer, eluted with Laemmli loading buffer, and analyzed by immunoblotting with the indicated antibodies directed against TRF2, BLM or Topo III (see Materials and Methods).
(0.09 MB PDF)
