# Supplementary material for: The G-Quadruplex Ligand Telomestatin Impairs Binding of Topoisomerase IIIα to G-Quadruplex-Forming Oligonucleotides and Uncaps Telomeres in ALT Cells
Source: PLoS One. 2009 Sep 9;4(9):e6919. doi: 10.1371/journal.pone.0006919 (PMC2732903; doi:10.1371/journal.pone.0006919)
Supplement: Figure S1 — Topo III/TRF2 complex is impaired in MRC5V1/YFP-Topo III cells treated with 2 µM telomestatin for 48 h. In contrast, Topo III/BLM complex is not modified by telomestatin treatment. Co-Immunoprecipitation was performed with D6 rabbit polyclonal antibody directed against Topo III as described previously [1]. Briefly, 2×10^7^ cells were lysed using RIPA buffer supplemented with 330 mM NaCl. The extract was precleared by Protein G Sepharose 4 Fast Flow and was mixed overnight with D6 anti-Topo III antibody. 100 µl of protein G-Sepharose beads were added to each sample, and beads were collected by centrifugation and washed three times with PBS buffer, eluted with Laemmli loading buffer, and analyzed by immunoblotting with the indicated antibodies directed against TRF2, BLM or Topo III (see Materials and Methods). (0.09 MB PDF) [file pone.0006919.s001.pdf]

Supplementary Material

Figure S1: Topo III/TRF2 complex is impaired in MRC5V1/YFP-Topo III cells treated with 2 µM telomestatin for 48h. In contrast, Topo III/BLM complex is not modified by telomestatin treatment. Co-Immunoprecipitation was performed with D6 rabbit polyclonal antibody directed against Topo III as described previously [1]. Briefly, $2x10^7$ cells were lysed using RIPA buffer supplemented with 330 mM NaCl. The extract was precleared by Protein G Sepharose 4 Fast Flow and was mixed overnight with D6 anti-Topo III antibody. 100 µl of protein G-Sepharose beads were added to each sample, and beads were collected by centrifugation and washed three times with PBS buffer, eluted with Laemmli loading buffer, and analyzed by immunoblotting with the indicated antibodies directed against TRF2, BLM or Topo III (see Materials and Methods).

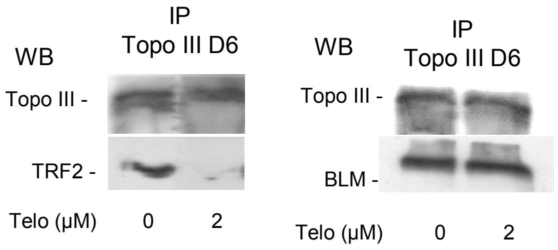

Figure S1

1. Temime-Smaali N, Guittat L, Wenner T, Bayart E, Douarre C, et al. (2008) Topoisomerase IIIalpha is required for normal proliferation and telomere stability in alternative lengthening of telomeres. Embo J 27: 1513-1524.
